# Association of Cumulative Lead Exposure with Parkinson’s Disease

**DOI:** 10.1289/ehp.1002339

**Published:** 2010-08-31

**Authors:** Marc G. Weisskopf, Jennifer Weuve, Huiling Nie, Marie-Helene Saint-Hilaire, Lewis Sudarsky, David K. Simon, Bonnie Hersh, Joel Schwartz, Robert O. Wright, Howard Hu

**Affiliations:** 1 Department of Environmental Health and; 2 Department of Epidemiology, Harvard School of Public Health, Boston, Massachusetts, USA; 3 Channing Laboratory, Department of Medicine, Harvard Medical School and Brigham and Women’s Hospital, Boston, Massachusetts, USA; 4 Rush University Institute for Healthy Aging, Rush University Medical Center, Chicago, Illinois, USA; 5 School of Health Sciences, Purdue University, West Lafayette, Indiana, USA; 6 Department of Neurology, Boston University Medical Center, Boston, Massachusetts, USA; 7 Department of Neurology, Brigham and Women’s Hospital, Boston, Massachusetts, USA; 8 Department of Neurology, Beth Israel Deaconess Medical Center and Harvard Medical School, Boston, Massachusetts, USA; 9 Harvard Vanguard Medical Associates, Boston, Massachusetts, USA; 10 Department of Pediatrics, Harvard Medical School, Children’s Hospital, Boston, Massachusetts, USA; 11 Department of Environmental Health Sciences, University of Michigan School of Public Health, Ann Arbor, Michigan, USA

**Keywords:** biomarker, bone lead, case–control study, epidemiology, humans, metals, risk factor

## Abstract

**Background:**

Research using reconstructed exposure histories has suggested an association between heavy metal exposures, including lead, and Parkinson’s disease (PD), but the only study that used bone lead, a biomarker of cumulative lead exposure, found a nonsignificant increase in risk of PD with increasing bone lead.

**Objectives:**

We sought to assess the association between bone lead and PD.

**Methods:**

Bone lead concentrations were measured using ^109^Cd excited K-shell X-ray fluorescence from 330 PD patients (216 men, 114 women) and 308 controls (172 men, 136 women) recruited from four clinics for movement disorders and general-community cohorts. Adjusted odds ratios (ORs) for PD were calculated using logistic regression.

**Results:**

The average age of cases and controls at bone lead measurement was 67 (SD = 10) and 69 (SD = 9) years of age, respectively. In primary analyses of cases and controls recruited from the same groups, compared with the lowest quartile of tibia lead, the OR for PD in the highest quartile was 3.21 [95% confidence interval (CI), 1.17–8.83]. Results were similar but slightly weaker in analyses restricted to cases and controls recruited from the movement disorders clinics only (fourth-quartile OR = 2.57; 95% CI, 1.11–5.93) or when we included controls recruited from sites that did not also contribute cases (fourth-quartile OR = 1.91; 95% CI, 1.01–3.60). We found no association with patella bone lead.

**Conclusions:**

These findings, using an objective biological marker of cumulative lead exposure among typical PD patients seen in our movement disorders clinics, strengthen the evidence that cumulative exposure to lead increases the risk of PD.

Parkinson’s disease (PD) is a complex disease for which a number of genetic and environmental risks have been identified. Twin studies suggest that environmental risk factors may be particularly important in patients whose illness begins after 50 years of age (Tanner et al. 1999). Although much of the research into environmental contributors to PD has focused on pesticides, other toxicants have been explored as well (Elbaz and Moisan 2008; Lai et al. 2002). Lead is known to disrupt dopaminergic function in experimental studies and can induce oxidative stress (Ercal et al. 2001), which is a candidate hypothesis for the etiology of PD (Jenner 2003). The assessment of exposure to lead, particularly cumulative exposure, however, can be difficult.

Lead can be measured easily in blood, but its half-life is approximately 30 days, rendering this biomarker a better indicator of recent exposure than of cumulative exposure. Cumulative exposure, however, might be more relevant than recent exposure for the development of PD. Prior studies of lead and PD have generally relied on self-reported exposure or on work histories from which cumulative exposures are reconstructed. Although these studies have suggested an association with cumulative exposure to lead, biomarkers of cumulative exposure could provide a more robust exposure measure. Circulating lead is deposited in bone, where it has a half-life on the order of years to decades, making it an excellent biomarker of cumulative lead exposure (Hu et al. 1998). Bone lead can be assessed with the K-shell X-ray fluorescence (KXRF) technique, but only one study has examined the association of this biomarker with PD, finding a suggestive but not quite significant [odds ratio (OR) = 1.62; 95% confidence intervals (CI), 0.83–3.17] association in an analysis involving 121 PD patients and 414 controls (Coon et al. 2006). We examined the association between cumulative exposure to lead—as measured by lead in bone with KXRF—and odds of PD in a case–control study based in Boston, Massachusetts.

## Materials and Methods

### Study population

Between 2003 and 2007, we recruited PD patients from four different movement disorders clinics in the Boston, Massachusetts, area: Boston University Medical Center (BUMC), Brigham and Women’s Hospital (BWH), Beth Israel Deaconess Medical Center (BIDMC), and Harvard Vanguard Medical Associates (HVMA). Cases from these four sites were confirmed by movement disorder specialists using the U.K. brain bank criteria (Hughes et al. 1992). In addition, the patients had to have two evaluations by a neurologist at least 6 months apart. We included additional cases from the Normative Aging Study (NAS), a Boston-based, longitudinal study of aging in men that was established in 1963 (Bell et al. 1966). From 1991 through 1999, 876 NAS participants had their bone lead measured with KXRF, as described previously (Weisskopf et al. 2007). Case ascertainment in the NAS was by self-report of having been diagnosed with PD by a physician—all NAS cases were taking PD medications.

Controls were recruited from among spouses, in-laws, and friends of the cases who had been recruited from the movement disorders clinics; through community advertisement; and from participants in the Harvard Cooperative Program on Aging (HCPOA) research participant cohort. The HCPOA is a program of the Hebrew SeniorLife Institute for Aging Research in Boston that maintains a registry of ethnically diverse elderly volunteers who have agreed to participate in research studies. We additionally included as a control any NAS participant without PD who was born in the same year as an NAS case and who had a bone lead measurement within 1 year of an NAS case. These restrictions were applied because there is a wide range of ages and years of bone lead measurements among men in the NAS cohort and because there are age and calendar year trends in bone lead levels (Kim et al. 1997).

We recruited a total of 344 PD patients and 324 controls. Fourteen PD patients and 16 controls did not have valid tibia bone lead measurements and were excluded; we included a total of 330 cases and 308 controls in the analyses. Of the cases, 163 were recruited from BUMC, 75 from BWH, 48 from BIDMC, 40 from HVMA, and 4 from the NAS cohort. Among the controls, 69 were recruited from among spouses, in-laws, and friends of the cases from the movement disorders clinics; 97 were NAS participants; and the remaining 142 controls were from the HCPOA or recruited through community advertisement. Only one control did not have a valid patella bone lead measurement, but six cases and five other controls did not have lead measurements taken at their patella. Thus, we included 338 cases and 318 controls in our analyses of patella bone lead and PD.

This study was approved by the Human Research Committee of the Harvard School of Public Health, the BWH, and the VA Boston Healthcare System. Written informed consent was obtained from all participants.

### Bone lead levels measured by KXRF

Bone lead measurements were taken at two anatomical sites with a KXRF instrument. When we began measuring bone lead, we used an instrument developed by ABIOMED (Danvers, MA). A technical description and validity specifications of this instrument have been published elsewhere (Aro et al. 2000; Burger et al. 1990; Hu et al. 1990). In 1999, we replaced our prototype ABIOMED instrument with an upgraded instrument designed to improve measurement precision, with changes in the cadmium radiation source, adjustments to the geometry of the measurement procedure, and upgrades in both the software and specific hardware components of the system (Aro et al. 1994). Thirty-minute measurements were taken of the left tibia and patella, after each region had been washed with a 50% solution of isopropyl alcohol. The tibia was measured at midshaft—the midpoint between the tibial plateau and the medial malleolus. The KXRF beam collimator was sited perpendicular to the flat bony surface of the tibia and at 30° in the lateral direction for the patella. Tibia and patella bone lead measurements with estimated uncertainties > 10 and 15 μg/g bone, respectively, were excluded, as described above, because these measurements usually reflect excessive subject movement during the measurement.

Tibia bone is primarily cortical bone, in which lead has a lower turnover rate—estimated at a half-life of > 20 years in previous studies (Kim et al. 1997), which makes it a good surrogate for lifetime exposure. In contrast, patella lead has a half-life of less than a decade, because it is trabecular bone, so these two exposure indices can help determine the relevant exposure interval for any association.

### Statistical analyses

We used logistic regression to estimate ORs of PD by bone lead concentration and their 95% CIs, adjusting for age, age squared, sex, race (white/other), pack-years of cigarette smoking, education (high school diploma or less, some college, college graduate, graduate school), and recruitment site. After accounting for age, age squared, sex, race, pack-years of smoking, and education, bone lead levels in the HCPOA and community controls were virtually the same as levels among the controls who were recruited from BUMC (tibia difference: −0.58 μg/g bone mineral, *p* = 0.79; patella difference: −0.90 μg/g bone mineral, *p* = 0.74). Thus, HCPOA and community controls were grouped with subjects recruited from BUMC. Cases and controls from the NAS had higher bone lead levels than did other participants in our study, which, because of the much larger control-to-case ratio among NAS participants compared with other recruitment sites in our steady, biases downward the crude association between bone lead and PD. The inclusion of the terms for recruitment site controls for this bias. For variables with missing data for some participants (never > 9% for any variable in any model), separate missing categories were entered into the models. Because the use of missing indicator categories can possibly introduce bias, we ran the same models using the multiple imputation procedure in SAS (PROC MI and MIANALYZE) to impute missing values and combine results from ten imputations as has been described elsewhere (Graham 2009). Results from these models differed little from our main analyses, so only the latter are presented. Bone lead was categorized into quartiles separately for each lead–PD model; thus, the numbers of cases and controls by quartile differ across models. Tests for trend were assessed by including a continuous term in the model created by assigning to each bone lead category the median of concentrations in that category. As sensitivity analyses, we also ran models using conditional logistic regression with stratification by recruitment site. The results of these models were similar to logistic regression models, so only the latter are presented. A spurious association between lead and risk of PD could result if lead is associated with duration of survival with PD. Therefore, we used linear regression to assess this association. In these analyses, we regressed duration of PD, in years, on bone lead and all other covariates. The threshold for statistical significance was set at *p* < 0.05. All analyses were conducted with SAS software (version 9; SAS Institute, Inc., Cary, NC).

## Results

The overall mean ± SD patella and tibia bone lead concentrations were 13.6 ± 15.9 and 10.7 ± 12.1 μg/g bone mineral, respectively. Table 1 shows the distribution of covariates among the cases, all controls from all sites with cases, and the subset of controls recruited from the movement disorders clinic sites. A majority of controls came from the NAS and HCPOA. Table 2 shows the distribution of covariates by tibia bone lead quartile among all controls. Older age, higher percentage of men, and lower educational attainment with increasing tibia lead quartile reflects in part the greater representation of NAS participants—who are all male, tend to be older with lower educational attainment, and have higher bone lead levels—among the controls.

Because we recruited no PD cases from the HCPOA or through community advertisement and therefore could not include a term for this group in our models to control for potential confounding introduced by unmeasured differences among this group, our primary analyses excluded these controls. In these analyses (Table 3), the odds of PD were significantly higher among those in the highest quartile of tibia bone lead than among those in the lowest (OR = 3.21; 95% CI, 1.17–8.83; *p* for trend = 0.02). In the analyses that were restricted to cases who were recruited from the movement disorders clinics and their controls (spouse/in-law/friend) (Table 3), we obtained similar results, with an OR of 2.57 (95% CI, 1.11–5.93; *p* for trend = 0.03) for PD among those in the highest versus the lowest quartile. In the analyses of all cases and controls, the results were also similar although slightly weaker (OR = 1.91; 95% CI, 1.01–3.60; *p* for trend = 0.06). This finding suggests some negative confounding that we could not control for because we recruited no cases from the HCPOA or through community advertisement. We found no association between patella bone lead and PD (Table 4).

We found no association between duration of PD and either patella bone lead (−0.48 years per SD increase in patella bone lead; *p* = 0.37) or tibia bone lead (0.12 years per SD increase in tibia bone lead; *p* = 0.79). The association between tibia bone lead concentration and PD was slightly stronger when we restricted cases to those with less than the median PD duration (5.5 years) than when we restricted it to those with more than the median PD duration. When we excluded HCPOA and community advertisement controls from the analyses, the OR for PD among those in the highest compared with lowest quartile was 3.34 (95% CI, 1.06–10.55) for the shorter duration cases and 2.81 (95% CI, 0.86–9.17) for the longer duration cases. We found little difference in results when we stratified by sex or by education (at least college graduate vs. less education; data not shown).

## Discussion

In this large case–control study with biomarker data on cumulative exposure to lead and more than twice as many cases as the only previous study with such biomarker data (Coon et al. 2006), we found increasing odds of PD with increasing tibia (cortical) bone lead, which has a half-life of decades (Kim et al. 1997). The positive association with tibia bone lead remained fairly consistent whether analyses excluded HCPOA and community advertisement controls, included only movement disorders clinic cases and their spouse/in-law/friend controls, or included all cases and controls. Our analyses adjusted for age, sex, race, smoking, and education; thus, the association with tibia lead appears to be independent of these factors. We found no association between patella (trabecular) bone lead and PD. The half-life of lead in trabecular bone is approximately 8 years, whereas that in cortical bone is several decades (Kim et al. 1997). Thus, cortical bone lead represents a cumulative exposure marker for lead exposure that is more long term than is trabecular bone lead. The lack of association between patella lead and PD therefore may suggest that the relevant exposure window for lead driving the association with PD is many years, even decades, before PD onset.

Although exposure to metals in general has been considered as a possible etiologic factor in the development of PD, few studies, other than a few case series and a case report, have specifically focused on lead (Kuhn et al. 1998; Sanz et al. 2007; Winkel et al. 1995). A case–control study in Belgium found 76% elevated odds of PD among those self-reporting lead exposure, but this was not significant (Pals et al. 2003). A case–control study within the Henry Ford Health System used occupational histories to estimate exposures to different metals and found significantly increased odds of PD with more than 20 years of combined exposure to lead and copper (OR = 5.24; 95% CI, 1.59–17.21) and lead and iron (OR = 2.83; 95% CI, 1.07–7.50) compared with those with no exposure (Gorell et al. 1997). A follow-up case–control study in the Henry Ford Health System assessed blood and tibia bone lead levels and also reconstructed exposures based on occupational history: Coon et al. (2006). Those authors created a model that combined all these measures to estimate lifetime lead exposure and found that, compared with those in the lowest quartile of this metric, those in the highest quartile had an OR of PD of 2.27 (95% CI, 1.13–4.55). When they considered only tibia bone lead, their results (OR = 1.62; 95% CI, 0.83–3.17) were less robust than those of our present study, possibly because of the smaller number of subjects in the earlier study. The mean concentrations of tibia lead were similar between the prior study and ours.

There are several mechanisms by which lead could be related to the development of PD. There is considerable experimental evidence that lead disrupts the dopaminergic system. Acutely, lead increases spontaneous dopamine release, inhibits depolarization-evoked dopamine release, decreases dopamine neuron spontaneous activity *in vivo*, and can alter dopamine-dependent behaviors (Cory-Slechta 1997; Minnema et al. 1986; Tavakoli-Nezhad et al. 2001). Studies have indicated that dopamine synthesis, turnover, and uptake in the midbrain and basal ganglia are decreased after lead exposure (Govoni et al. 1979; Jadhav and Ramesh 1997; Lasley 1992; Lucchi et al. 1981; Missale et al. 1984), as is dopamine D_1_/D_2_ receptor expression (Gedeon et al. 2001). In addition, excessive oxidative toxicity is a candidate etiologic factor for PD (Jenner 2003), and lead exposure is a well-known prooxidant, inducing oxidative stress through direct actions on cell membranes, interactions with hemoglobin or δ-aminolevulinic acid dehydratase, or depletion of antioxidant defenses (Ercal et al. 2001).

A strength of our study is the use of bone lead measurements as biomarkers of cumulative lead exposure over many years. A limitation of this study is that we measured these levels several years after the diagnosis of PD, leading to the possibility that the disease state could affect the bone lead levels. However, we used bone lead levels as the biomarker rather than blood lead levels, and the long half-life of lead in bone means that the assessment of exposure even several years after diagnosis will still reflect to some extent exposures prior to disease diagnosis. Furthermore, it is unlikely that external lead exposures would become higher among PD patients after disease diagnosis. If lead exposure caused longer survival with PD, then this could bias our results, but this seems unlikely, and bone lead was not associated with PD duration in our data.

Having bone lead measurements from both tibia (cortical) and patella (trabecular) bone is an additional strength of our study that helps to address the concern that osteoporosis and osteopenia, which are common in PD (Invernizzi et al. 2009), could differentially affect the lead levels measured in cases and controls and bias the results. Although osteoporosis and osteopenia are the result of bone loss, the most important factor influencing bone lead content is the rate of bone formation rather than the rate of bone resorption (Hu et al. 1998). As bone is resorbed, the lead concentration in the remaining bone (on a per gram of bone mineral basis) should remain the same. If new bone formation were to slow more in PD patients than in controls, then PD patients would be forming less new bone in recent years compared with controls—and because external exposures to lead have been much lower in recent years than in the past, one would expect the overall concentration of lead in bone to decline less in the cases than in controls. However, because bone lead appears to decay much more slowly in cortical bone than in trabecular bone during aging, increased odds of PD as a result of bias from faster reductions in bone lead among controls (because of relatively greater new bone formation) compared with cases would be expected to be more pronounced for patella bone lead than for tibia bone lead. Our findings show no association between patella bone lead and PD and therefore suggest that this potential bias did not account for the association between tibia lead and PD. Furthermore, if this potential bias explained our results, then we would expect the association to be stronger for cases with longer PD duration, which was not the case.

An additional limitation is that our recruitment process for both cases and controls was such that we cannot determine the participation rates for our study. The recruitment of controls by several distinct approaches could raise concern that one set of controls might drive the results, but arguing against this is the observation that our results were similar among different subsets of controls. It is possible that differences in bone lead measurements could be introduced by the use of two different KXRF machines, but because one machine was used exclusively on both cases and controls from the NAS, the inclusion of a term for NAS participants in our models as part of our adjustment for recruitment site would control for any such measurement differences by machine.

The use of spouse, in-law, and friend controls can result in controls being more similar to cases for some variables—importantly, lead exposure—than would be found in the general target population. This similarity may explain the association between smoking and PD in our study—an adjusted OR per 10 pack-years of 0.86 (95% CI, 0.71–1.04), which is not as strong an inverse association as in other reports (Hernan et al. 2002), likely because smoking behaviors tend to be concordant among spouses, relatives, and friends (Di Castelnuovo et al. 2009). As our smoking results illustrate, this bias is almost always toward the null; thus, if it also affects the results for lead exposure, our results would be more likely to be muted rather than spuriously large. In addition, we recruited most of the cases in our study from movement disorders clinics, and there tend to be differences between PD cases seen at clinics compared with a community sample of cases. PD cases seen in clinics have been reported to more likely be, for example, younger and male (Rybicki et al. 1995). These differences would be unlikely to introduce any referral bias in our study because we adjusted for these factors. However, clinic PD cases have also been reported to be more likely to have private health insurance, to be frequent users of health care, and to use hospital-based clinics for their primary health care (Rybicki et al. 1995). If these characteristics are also associated with lead exposure, bias could be introduced into our results. Indeed, if these characteristics are associated with lower lead exposure, then this referral bias may partly explain the attenuated results we found when including HCPOA and community controls.

In summary, in this large case–control study, we found evidence that higher cumulative exposure to lead is associated with an increased risk of PD. Our data provide some of the strongest evidence to date for a role for lead in the development of PD.

## Figures and Tables

**Table 1:** Baseline characteristics by case–control status of all participants with tibia bone lead measurements.

Characteristics	Cases (*n* = 330)	All controls (*n* = 308)	Controls from sites with cases (*n* = 166)	Controls from movement disorders clinics (*n* = 69)
Age (years) at KXRF (mean ± SD)	66.5 ± 9.5	69.4 ± 8.8	68.8 ± 7.2	65.4 ± 9.0
Years since diagnosis (mean ± SD)	6.9 ± 5.8	NA	NA	NA
Pack-years, median (25th–75th percentile)	0 (0–8)	0.8 (0–17.6)	2.3 (0–19.5)	0 (0–6.5)
Male [*n* (%)]	216 (65.5)	172 (55.8)	126 (75.9)	29 (42.0)
Nonwhite race [*n* (%)]	35 (10.6)	52 (16.9)	15 (9.0)	14 (20.3)
Education [*n* (%)]				
≤ High school graduate	45 (13.6)	74 (24.0)	51 (30.7)	6 (8.7)
Some college or trade school	42 (12.7)	70 (22.7)	39 (23.5)	15 (21.7)
College graduate	97 (29.4)	78 (25.3)	35 (21.1)	17 (24.6)
Postgraduate	123 (37.3)	70 (22.7)	28 (16.9)	19 (27.5)
Missing	23 (7.0)	16 (5.2)	13 (7.8)	12 (17.4)
Recruitment site [*n* (%)]				
BUMC	163 (49.4)	39 (11.0)	39 (23.5)	39 (56.5)
BWH	75 (22.7)	13 (3.7)	13 (7.8)	13 (18.8)
BIDMC	48 (14.6)	4 (1.1)	4 (2.4)	4 (5.8)
HVMA	40 (12.1)	13 (3.7)	13 (7.8)	13 (18.8)
NAS	4 (1.2)	97 (27.4)	97 (58.4)	0
HCPOA	0	135 (38.1)	0	0
Community	0	7 (2.0)	0	0

NA, not applicable.

**Table 2:** Baseline characteristics of controls by quartile^a^ of tibia bone lead.

	Quartile			
Characteristic	First (*n* = 74)	Second (*n* = 83)	Third (*n* = 74)	Fourth (*n* = 77)
Tibia lead [μg/g bone mineral]	≤ 5	5.2–10.4	11.0–19.0	≥ 19.1
Age (years) at KXRF (mean ± SD)	64.4 ± 11.2	70.5 ± 8.2	71.0 ± 7.1	71.7 ± 6.2
Pack-years, median (25th–75th percentile)	0 (0–3.7)	0 (0–11.0)	6.3 (0–18.9)	6.3 (0–28.3)
Male [*n* (%)]	22 (29.7)	42 (50.6)	46 (62.2)	62 (80.5)
Nonwhite race [*n* (%)]	22 (29.7)	14 (16.9)	10 (13.5)	6 (7.8)
Education [*n* (%)]				
≤ High school graduate	8 (10.8)	11 (13.3)	14 (18.9)	41 (53.3)
Some college or trade school	13 (17.6)	16 (19.3)	24 (32.4)	17 (22.1)
College graduate	19 (25.7)	29 (34.9)	18 (24.3)	12 (15.6)
Postgraduate	26 (35.1)	22 (26.5)	17 (23.0)	5 (6.5)
Missing	8 (10.8)	5 (6.0)	1 (1.4)	2 (2.6)

a Different from quartiles used in some of the models (see “Materials and Methods”).

**Table 3:** Adjusted^a^ OR for PD by quartile of tibia bone lead.

	All cases, and spouse/in-law/friend and NAS controls^b^			Movement disorders clinic cases only, and only spouse/in-law/friend controls^c^			All cases and controls		
Quartile	Lead (μg/g)	Cases/controls	OR (95% CI)	Lead (μg/g)	Cases/controls	OR (95% CI)	Lead (μg/g)	Cases/controls	OR (95% CI)
First	< 3.1	90/30	Reference	< 1	68/24	Reference	< 3.1	90/66	Reference

Second	3.5–9.6	101/31	1.36 (0.70–2.63)	1.7–7.0	86/18	1.48 (0.72–3.04)	3.5–9.0	92/64	1.30 (0.76–2.23)

Third	10.0–17.0	85/33	1.90 (0.90–4.01)	7.8–13.1	88/16	1.91 (0.91–4.00)	9.6–16.0	91/76	1.37 (0.80–2.36)

Fourth	> 17.3	54/72	3.21 (1.17–8.83)	> 13.9	84/11	2.57 (1.11–5.93)	> 16.0	57/102	1.91 (1.01–3.60)

*p*-Trend			0.02			0.03			0.06

a Adjusted for age, age squared, sex, race, pack-years of cigarette smoking, education, and recruitment site.

b Excluding HCPOA and community advertisement controls.

c Excluding NAS cases and NAS, HCPOA, and community advertisement controls.

**Table 4:** Adjusted^a^ OR for PD by quartile of patella bone lead.^b^

	All cases, and spouse/in-law/friend and NAS controls^c^			Movement disorders clinic cases only, and only spouse/in-law/friend controls^d^			All cases and controls		
Quartile	Lead (μg/g)	Cases/controls	OR (95% CI)	Lead (μg/g)	Cases/controls	OR (95% CI)	Lead (μg/g)	Cases/controls	OR (95% CI)
First	< 2.7	101/29	Reference	< 1	77/21	Reference	< 2.7	101/62	Reference

Second	3.5–11.0	89/32	0.98 (0.50–1.93)	1.7–7.8	86/24	1.04 (0.51–2.14)	3.5–11.0	89/74	1.12 (0.66–1.91)

Third	11.3–20.9	100/35	1.71 (0.81–3.62)	8.7–15.7	83/14	1.44 (0.64–3.29)	11.3–20.0	94/69	1.37 (0.80–2.39)

Fourth	> 20.9	48/77	1.15 (0.45–2.93)	> 16.5	88/17	1.20 (0.52–2.76)	> 20.0	54/113	1.03 (0.54–1.95)

*p*-Trend			0.42			0.47			0.83

a Adjusted for age, age squared, sex, race, pack-years of cigarette smoking, education, and recruitment site.

b Because of availability of valid patella bone lead measurements, as described in “Materials and Methods,” eight more cases and six fewer controls in the total sample had patella bone lead analyses than had tibia bone lead analyses.

c Excluding HCPOA and community advertisement controls.

d Excluding NAS cases and NAS, HCPOA, and community advertisement controls.
